# Optimising breast cancer screening reading: blinding the second reader to the first reader’s decisions

**DOI:** 10.1007/s00330-021-07965-z

**Published:** 2021-06-12

**Authors:** Jennifer A. Cooper, David Jenkinson, Chris Stinton, Matthew G. Wallis, Sue Hudson, Sian Taylor-Phillips

**Affiliations:** 1 Department of Health Sciences, Warwick Medical School, University of Warwick, Gibbet Hill Road, Coventry, CV4 7AL UK; 2 Population Health Sciences; Bristol Medical School, University of Bristol, Bristol, BS8 2BN UK; 3 Cambridge Breast Unit, Cambridge University Hospitals National Health Service Foundation Trust, and National Institute for Health Research Cambridge Biomedical Research Centre, Cambridge, UK; 4 Peel & Schriek Consulting Limited, London, UK; 5 Warwick Screening, Warwick Medical School, University of Warwick, Coventry, CV4 7AL UK

**Keywords:** Breast neoplasms, Mammography, Mass screening, Early detection of cancer, Markov chains

## Abstract

**Objectives:**

In breast cancer screening, two readers separately examine each woman’s mammograms for signs of cancer. We examined whether preventing the two readers from seeing each other’s decisions (blinding) affects behaviour and outcomes.

**Methods:**

This cohort study used data from the CO-OPS breast-screening trial (1,119,191 women from 43 screening centres in England) where all discrepant readings were arbitrated. Multilevel models were fitted using Markov chain Monte Carlo to measure whether reader 2 conformed to the decisions of reader 1 when they were not blinded, and the effect of blinding on overall rates of recall for further tests and cancer detection. Differences in positive predictive value (PPV) were assessed using Pearson’s chi-squared test.

**Results:**

When reader 1 recalls, the probability of reader 2 also recalling was higher when not blinded than when blinded, suggesting readers may be influenced by the other’s decision. Overall, women were less likely to be recalled when reader 2 was blinded (OR 0.923; 95% credible interval 0.864, 0.986), with no clear pattern in cancer detection rate (OR 1.029; 95% credible interval 0.970, 1.089; Bayesian *p* value 0.832). PPV was 22.1% for blinded versus 20.6% for not blinded (*p* < 0.001).

**Conclusions:**

Our results suggest that when not blinded, reader 2 is influenced by reader 1’s decisions to recall (alliterative bias) which would result in bypassing arbitration and negate some of the benefits of double-reading. We found a relationship between blinding the second reader and slightly higher PPV of breast cancer screening, although this analysis may be confounded by other centre characteristics.

**Key Points:**

*• In Europe, it is recommended that breast screening mammograms are analysed by two readers but there is little evidence on the effect of ‘blinding’ the readers so they cannot see each other’s decisions.*

*• We found evidence that when the second reader is not blinded, they are more likely to agree with a recall decision from the first reader and less likely to make an independent judgement (alliterative error). This may reduce overall accuracy through bypassing arbitration.*

*• This observational study suggests an association between blinding the second reader and higher positive predictive value of screening, but this may be confounded by centre characteristics.*

**Supplementary Information:**

The online version contains supplementary material available at 10.1007/s00330-021-07965-z.

## Introduction

Breast cancer screening is implemented in many European countries. European quality assurance guidelines recommend that mammograms are examined for signs of cancer by two radiologists (readers) using two mammographic views [1, 2]. There is evidence that this approach increases the cancer detection rate compared to single reading [3–7]. A retrospective analysis of women participating in the English NHS Breast Screening Programme identified that double reading with arbitration of discordant decisions reduced recall and increased cancer detection rates, compared to hypothetical single reading [7]. However, the cancers detected only by the second reader were smaller, had fewer involved nodes, and were of lower grade [7]. This finding is consistent with some prior research [8]. The identification of smaller lower grade cancers and DCIS may be beneficial, or it may not be a desirable outcome of breast cancer screening due to their association with overdiagnosis [9]. There is therefore currently debate about the efficacy of double reading [10].

An aspect of double reading that has received little research to date is the blinding of reader 2 to the decisions of reader 1. Previous European guidance has recommended blinding, but the most recent version omits any recommendation on blinding except for in research studies [1, 2]. There is some evidence that blinding may affect diagnostic accuracy and patient outcomes. One study investigated a consecutive series of mammograms from women participating in the national Dutch screening programme, with no arbitration of discordant results. This study found that blinded double reading resulted in higher programme sensitivity than non-blinded reading (83% vs 76%, *p* = 0.01) [11], albeit with higher benign biopsy rates when blinded (2.6 vs 1.4 per 1000 screens *p* < 0.001 for ultrasound-guided core needle biopsy (CNB), and 5.9 vs 4.7 per 1000 screens *p* = 0.013 for stereotactic CNB) [12]. These results suggest that reader 2 might be influenced by reader 1’s decisions, but are not applicable to screening programmes which use arbitration of discordant reader decisions. The same study team produced some projections of the effect of blinding in this context, using a retrospective laboratory rather than clinical practice arbitration decision [13, 14].

In his monograph on errors in radiology, Smith [15] introduced the term ‘alliterative error’ to describe the influence that one radiologist can have on another. He suggested that, for example, if during an initial interpretation of a radiographic image an abnormality is missed, or a benign finding overemphasised, subsequent interpretations may lead to the same erroneous conclusion. This can occur when the subsequent interpretation of the original image is carried out by a different reader or the original reader. Smith proposed that this may occur because the second reader reads the results of previous examinations before making their own decision, and then tend to adopt the same position, conforming to the belief of their peers. While there have been few published studies of alliterative errors, it is often reported as a source of error in radiology [16–19].

If non-blinded decision-making can introduce alliterative bias, this could affect rates of recall, cancer detection, and outcomes for women attending screening. Optimising reading conditions could improve the balance of benefits and harms of breast screening. The aim of this research was to determine the effect of blinding the second reader in breast cancer screening on alliterative error and subsequently the effect on screening accuracy (recall and cancer detection rate), in a population breast screening programme which uses arbitration of discrepant reader decisions.

## Materials and methods

### Study design

This study is reported using the ‘STROBE’ statement [20]. The study is a population-based cohort study within the Changing Case Order to Optimise Patterns of Performance in Screening (CO-OPS) trial. The original trial investigated patterns of performance and fatigue with time on task, and is described in detail elsewhere [21]. Briefly, the trial included 1,194,147 women (predominantly aged 47–73 years) attending routine triennial digital mammography screening between December 2012 and November 2014 at 46 English centres. Women with high-familial risk and who presented symptomatically were excluded. Digital mammograms were assessed independently by two expert readers (radiologists, radiography advanced practitioners, breast clinicians) for signs of cancer and whether a woman should be recalled for further investigation. Readers in the screening programme are required to examine a minimum of 5000 mammograms a year and have undergone extensive training [22]. Arbitration was used at all centres when there were disagreements between the two readers (13 centres used a third reader, 33 used group consensus of 2 or more readers). Additionally, at some centres, arbitration was used even when both readers suggested recall, in an effort to reduce overall recall rates. The National Breast Screening Service (NBSS) database records the decisions of the readers and clinical information for each woman.

### Data collection

Data were extracted from the NBSS system. Fields which indicate the ‘blind’ status at the time reader 1 and reader 2 saved their opinions were extracted. Reader 1 selects whether reading is blinded and then the second reader can change this during their reading session. When the blind reporting option is selected in NBSS, it masks the opinions of the previous reader by showing ‘Entered’ in place of the opinions. In the blinded reading condition, reader 2 could still ascertain what reader 1 decided by looking through the paper notes; however, this would be rare due to time constraints in the high-volume screening environment.

### Statistical analysis

Summary statistics of the characteristics of the women screened and the outcomes by the first reader, second reader and after arbitration of discordant decisions were presented by whether reader 2 was blinded. To investigate whether alliterative bias was present, we compared the proportion of cases where there were discordant decisions between readers using a chi-squared test. The hypothesis was that blinding the second reader would increase disagreements by reducing alliterative bias. We then directly modelled whether the second reader was influenced by the first reader’s decision when not blinded, i.e. whether alliterative bias was present. The model outcome was the second reader decision, with fixed effects for whether reader 1 recalled the woman and whether reader 2 was blinded, and an interaction term between them.

We fitted a multi-level model using Markov chain Monte Carlo (MCMC) methods using R2MLwiN [23], which runs the multilevel modelling program MLwiN [24, 25] from within the ‘R’ environment. A MCMC approach provides several advantages over maximum likelihood estimation in this context. It can achieve more accurate model estimates particularly with more complex models and gives a posterior probability distribution for the parameters, rather than a *p* value [26–28]. The unit of analysis was the woman screened, with clustering by reader and centre. We included fixed effects for whether a woman was attending her first or a subsequent screen and the woman’s age (continuous, centred). Random effects were included for the second reader (level 2) and screening centre (level 3).

To investigate whether any alliterative bias may affect screening accuracy, we modelled whether blinding the second reader was associated with differences in overall recall and cancer detection rates. Two interaction terms were considered for inclusion, based on the Bayesian deviance information criterion (DIC) to assess overall model fit and the *p* value of the z-score for an estimate (5% level) [23]. An interaction between blinding and age was included because younger women tend to have a higher density of breast tissue, increasing task difficulty [29]. An interaction between blinding and previous screening attendance was assessed because a lack of previous mammograms for comparison also increases task difficulty. Cancer detection and recall rate for reader 2 (without arbitration) were also modelled to assess the intervention effect (Supplementary Material Appendix A).

Tumour characteristics (DCIS grade, disease grade, invasive disease presence, number of positive axillary nodes, maximum diameter of invasive disease) were determined for blinded/non-blinded reader 2 with statistical testing (χ^2^ test for independence, test for equality of two proportions and t test) to determine any significant differences. The positive predictive value (PPV) when blinding the second reader compared to not blinding was reported, using the reference standard of biopsy-proven cancer after recall from screening. Pearson’s chi-squared test was used to compare PPV in cases read blinded and not blinded. To assess the potential impact of centre confounding (fully blinded centre, vs fully non-blinded, vs mixed centres), all the above models were run with a subset of six centres which had a mix of blinded and non-blinded reading as a sensitivity analysis. A mixed protocol centre was one where there was at least 5% of blinded or not blinded out of the total number of mammograms read at the centre (Supplementary Material Appendix C).

Interval cancers within 3 years of screening were used to estimate test accuracy metrics for blinded/non-blinded reading (sensitivity, specificity, PPV, negative predictive value (NPV)). We separated the women not recalled into ‘false negatives’ (women not recalled who had an interval cancer within 3 years of screening) and ‘true negatives’ (women not recalled and either did not have an interval cancer recorded in their follow-up data or did not have follow-up data). For consistency within this analysis, anyone recalled, had no cancer detected, and had an interval cancer within 3 years of screen was classified as a true positive, rather than a false positive. We performed an equality of proportions test to determine whether these were statistically significant (Supplementary Material Appendix E).

## Results

### Descriptive statistics

**Fig. 1:**
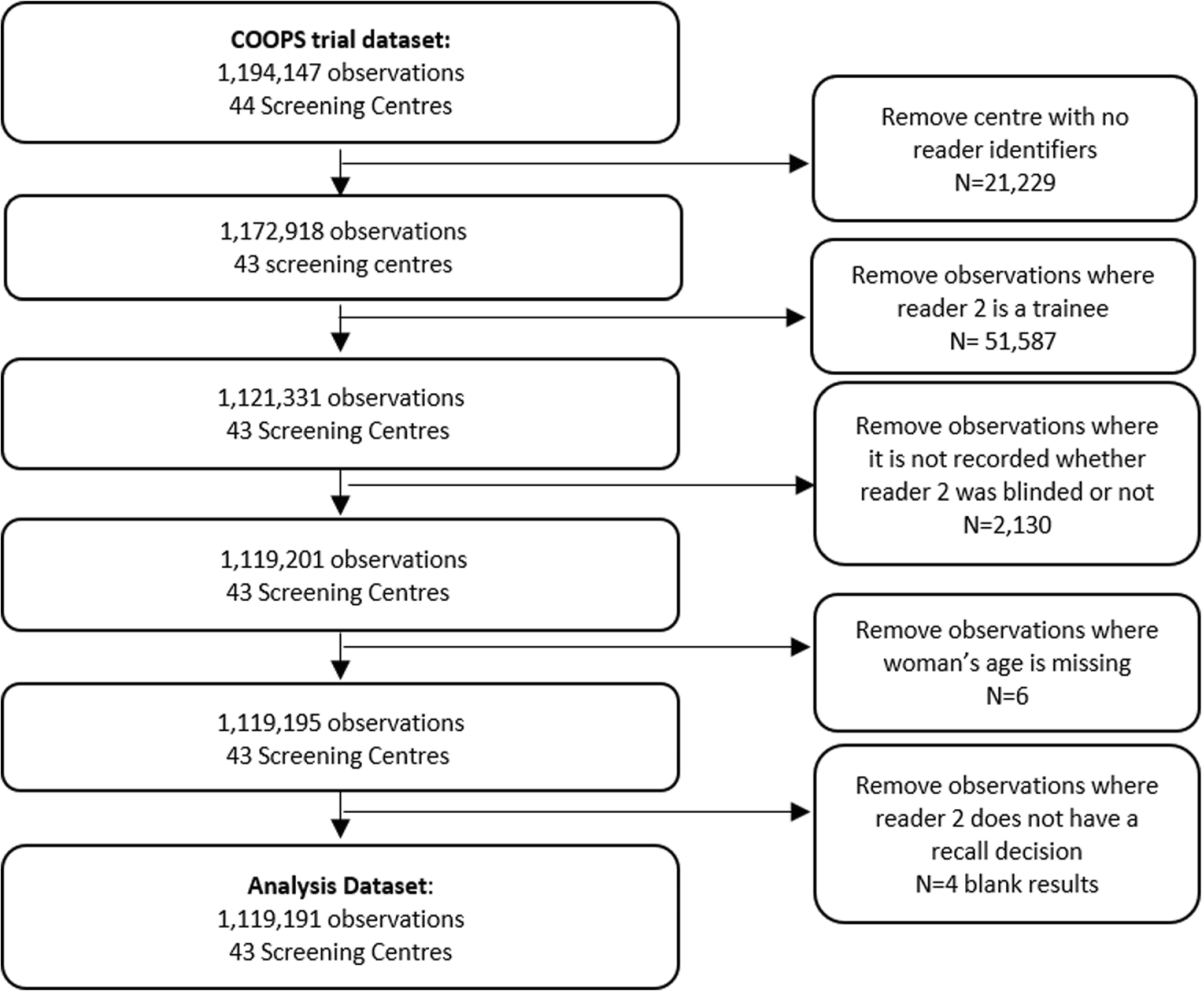
Study flow diagram with reasons for exclusions. There were 46 centres in the CO-OPS trial, but three shared a common computer system so are counted as one centre in this analysis, a further centre was removed which had no reader identifiers, giving 43 centres in the dataset. Of the 43 centres, 23 centres were classified as not blinded, 14 as blinded, and 6 as mixed. Reader 2 was blinded for 34.2% (382,490/1,119,191) of women screened

A total of 1,119,191 women were included from 43 screening centres with 9656 cancers detected after arbitration (0.86%). The mean age of the women was 59, and 78.8% had previously attended screening (881,900/1,119,191). The study flow diagram is depicted in Fig. 1. Study characteristics and outcomes by blinding status are presented in Table 1. Of the 43 centres, 23 centres were classified as not blinded, 14 as blinded, and 6 as mixed. There were 418 first readers and 420 second readers. Reader 2 was blinded for 34.2% of women screened.

**Table 1:** Characteristics of the study sample, and recall and cancer detection rates for blinded versus not blinded reader 2

Study characteristic		Reader 2 Blinded	%	Reader 2 Not blinded	%
Age of woman (mean)	Mean age	59.2	**-**	59.3	**-**
	Group 1 (52 or less)	90,505	23.66	167,217	22.70
	Group 2 (53-59)	111,642	29.19	214,996	29.18
	Group 3 (60 or more)	180,343	47.15	354,488	48.12
	Total	382,490	100.00	736,701	100.00
First screen/subsequent screen	Subsequent screen	300,820	78.65	581,080	78.88
	First screen	81,670	21.35	155,621	21.12
	Total	382,490	100.00	736,701	100.00
Reader 1 Recall (pre arbitration)	Not recalled	363,034	94.91	697,294	94.65
	Recalled	19,456	5.09	39,407	5.35
	Total	382,490	100.00	736,701	100.00
Reader 2 Recall (pre arbitration)	Not recalled	364,387	95.27	698,512	94.82
	Recalled	18,103	4.73	38,189	5.18
	Total	382,490	100.00	736,701	100.00
Recall (after arbitration)	Not recalled	367,341	96.04	706,082	95.84
	Recalled	15,149	3.96	30,619	4.16
	Total	382,490	100.00	736,701	100.00
Cancers detected by reader 1	Cancer detected	3066	0.80	5717	0.78
	No Cancer detected	379,424	99.20	730,984	99.22
	Total	382,490	100.00	736,701	100.00
Cancers detected by reader 2	Cancer detected	3226	0.84	6117	0.83
	No cancer detected	379,264	99.16	730,584	99.17
	Total	382,490	100.00	736,701	100.00
Cancers detected overall (after arbitration)	Cancer detected	3355	0.88	6301	0.86
	No Cancer detected	379,135	99.12	730,400	99.14
	Total	382,490	100.00	736,701	100.00

### Alliterative bias

Rates of disagreement between the two readers for recall were 0.20% points higher when blinded (3.57%; 95% CI: 3.51%, 3.63%) than when not (3.37%; 95% CI: 3.33%, 3.41%) (χ^2^(1) = 32.46, *p* < 0.001). The disagreement rate difference increases to 5.60% points when the first reader recalls the case (38.61%; 95% CI: 37.92%, 39.29%) when reader 2 is blinded versus (33.01%; 95% CI: 32.55%, 33.48%) when reader 2 is not blinded (χ^2^(1) = 179.03, *p* < 0.001) (Supplementary Table B.2).

**Fig. 2:**
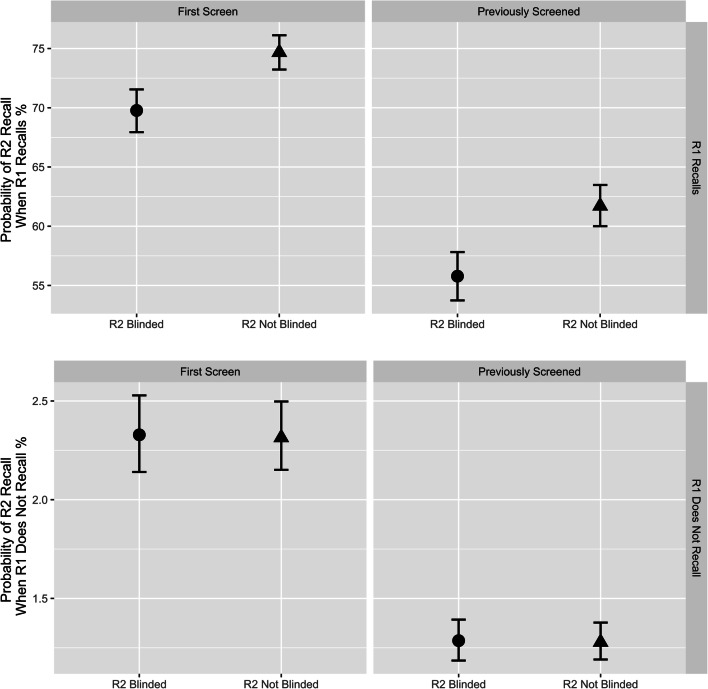
Facet plot showing the probability (with 95% credible interval) of reader 2 recalling a woman (at the mean age of 59.27) by whether reader 1 (R1) recalls or not, when reader 2 (R2) is blinded and not blinded. Results also presented for women who are first time screens or subsequent screens. The probability of recall is lower for a woman attending a subsequent screen compared to attending a first-time screen

The multilevel model results show a similar pattern (Fig. 2). When reader 1 recalls, the probability of reader 2 recalling is 4.9% points lower when blinded (69.8%; 95% credible interval: 67.9%, 71.5%) versus not blinded (74.7%; 95% credible interval: 73.2%, 76.1%) for a woman’s first screen. If reader 1 does not recall, the probability of reader 2 recalling when blinded (2.33%; 95% credible interval: 2.14, 2.53%) and not blinded (2.32%; 95% credible interval: 2.15, 2.50%) is similar for a woman’s first screen. The model and full probabilities are reported in Supplementary Tables B.1 and B.3.

### Recall rate, cancer detection rate, and PPV

#### Recall rate

Reader 2 (before arbitration) recalled 0.45% points fewer women when blinded (4.73%; 95% CI: 4.67%, 4.80%) than when not blinded (5.18%; 95% CI: 5.13%, 5.23%) (χ^2^(1) = 107.04, *p* < 0.001). However, reader 1 (who cannot see reader 2’s decision, as by definition it does not yet exist) also recalled fewer women (0.26% points) when reader 2 was blinded (5.09%; 95% CI: 5.02%, 5.16%) than when not blinded (5.35%; 95% CI: 5.30%, 5.40%) (χ^2^(1) = 34.751, *p* < 0.001) indicating at least part of this effect may be due to confounding (Table 1).

**Fig. 3:**
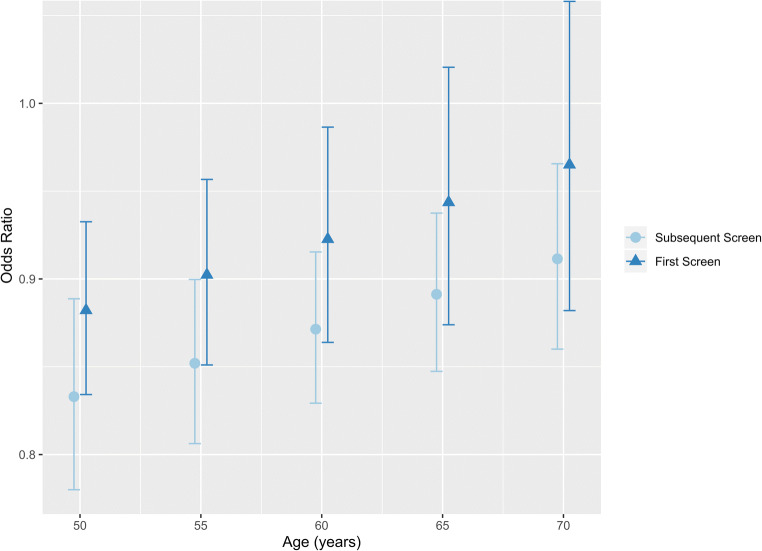
Interaction plot displaying the odds ratios (median and 95% credible intervals) of recall rate after arbitration when reader 2 is blinded versus not blinded by screen status and age. See accompanying Table A.2 in the Supplementary Material

Recall rate after arbitration was lower when reader 2 was blinded (3.96%; 95% CI: 3.90%, 4.02%) compared to when they were not (4.16%; 95% CI: 3.93%, 4.38%) (χ^2^(1) = 24.51, *p* < 0.001). A model assessing the effect of blinding reader 2 on the recall rate after arbitration is presented in Table 2 and Fig. 3. Blinding reader 2 decreased the odds of recall from screening compared to not blinding (OR 0.869; 95% credible interval 0.826, 0.913) for a woman of average age (59 years) who has previously been screened (Table 2).

**Table 2:** Markov chain Monte Carlo (MCMC) multilevel model determining the effect of blinding on recall rate overall (after arbitration). The full model including one-sided Bayesian *p* values is reported in the Supplementary Material (Table A.1) along with the caterpillar plot showing level 3 residuals and their 95% CIs (Figure A.1)

Recall rate overall (after arbitration) multilevel model			
Fixed Effects	Odds ratio^a^	95% credible interval for the odds ratio^b^	Pr(>|z|)^c^
Blinding Yes (versus no as the reference category)	0.869	0.826, 0.913	< 0.001
Age (centred)	1.007	1.005, 1.009	< 0.001
First screen (versus subsequent screen as the reference category)	2.823	2.728, 2.922	< 0.001
Blinded yes * age (interaction term)	1.005	1.001, 1.008	0.015
Blinding yes * first screen (interaction term)	1.060	0.999, 1.125	0.057

^**a**^The mean of the 100,000 chain iterations after converting from the log odds scale to the odds scale.

^**b**^95% credible interval is generated by taking the 2.5th and 97.5th quantiles of the 100,000 chain iterations after converting from the log odds scale to the odds scale

^**c**^Two-tailed *p* value of the z score for the coefficient (testing whether the estimate is significantly different from zero assuming normality)

The interactions for the recall rate model were dissected in an interaction plot (Fig. 3). Blinding reader 2 decreased the odds of recall after arbitration for both first time and subsequent screens, and for all ages. The trend was towards a greater effect of blinding on recall rate at younger ages, and when the woman had previously attended screening. For both first and subsequent screen mammograms of 60-year-old women, women were less likely to be recalled if reader 2 was blinded than if they were not: first screen OR 0.923 (95% credible interval 0.864, 0.986), subsequent screen OR 0.871 (95% credible interval 0.829, 0.915) (Fig. 2, Table A.2 Supplementary Material).

Analysis of the subset of 179,573 women at the six centres in which there was a mixture of blinded/unblinded second readers showed similar results. Blinding the second reader was associated with a lower recall rate after arbitration than when the second reader was not blinded (OR 0.883; 95% credible interval 0.834, 0.933) (Supplementary Table C.2).

#### Cancer detection rate

Reader 2 (before arbitration) detected cancers in 0.84% (95% CI: 0.81%, 0.87%) of women when blinded versus 0.83% (95% CI: 0.81%, 0.85%) when not blinded (χ^2^(1) = 0.51, *p* = 0.48). The cancer detection rate overall (with arbitration) was similar when reader 2 was blinded (0.88%; 95% CI: 0.85%, 0.91%) compared to when not (0.86%; 95% CI: 0.83%, 0.88%, χ^2^(1) = 1.4, *p* = 0.2).

The model determining the association of blinding with cancer detection rate after arbitration is reported in Table 3. The association between blinding and cancer detection was not statistically significant (OR 1.029; 95% credible interval: 0.970, 1.089; *p* = 0.341), although the Bayesian *p* value suggests that 83.2% of estimates lie above an odds ratio of 1 (showing a potential positive association) (Supplementary Material Table A.5). Cancer detection also increases with age and with a first screen versus a subsequent screen.

**Table 3:** Markov chain Monte Carlo (MCMC) multilevel model determining the effect of blinding on cancers detected overall (after arbitration). Interactions were not included for this model due to both a higher deviance information criterion (DIC) in comparison to a model without and non-significant *p* values for the estimates. The full model including one-sided Bayesian *p* values is reported in the Supplementary Material (Table A.5) along with the caterpillar plot showing level 3 residuals and their 95% CIs (Figure A.3)

Cancer detection overall (after arbitration) multilevel model			
Fixed effects	Odds ratio^a^	95% credible interval for the odds ratio^b^	Pr(>|z|)^c^
Blinding yes (versus no as the reference category)	1.029	0.970, 1.089	0.341
Age (centred)	1.052	1.049, 1.056	< 0.001
First screen (versus subsequent screen as the reference category)	1.696	1.591, 1.807	< 0.001

^**a**^The mean of the 100,000 chain iterations after converting from the log odds scale to the odds scale

^**b**^95% credible interval is generated by taking the 2.5th and 97.5th quantiles of the 100,000 chain iterations after converting from the log odds scale to the odds scale

^**c**^Two-tailed *p* value of the z score for the coefficient (testing whether the estimate is significantly different from 0 assuming normality)

Analysis of the subset of six centres (179,573 women) where there is a mix of blinded/unblinded second readers showed similar results (Supplementary Table C.4).

#### Tumour characteristics

Tumour characteristics by whether reader 2 is blinded or not is shown in Supplementary Table D.1. Invasive disease was present in 78.4% (2570/3277) for blinded and 76.6% (4503/5881) for not blinded (χ^2^(1) = 4.0, *p* = 0.0449); with no significant evidence of any difference for disease grade (χ^2^(2) = 0.67, *p* = 0.7), the number of positive axillary nodes (χ^2^(2) = 3.0, *p* = 0.2), and the mean diameter of the tumour for blinded (16.5 mm, standard deviation (SD) 12.6 mm) and not blinded (16.2 mm, SD 11.8 mm; t = 1.0, *p* = 0.3). When invasive disease was not present, there was no evidence of an effect of blinding on grade of DCIS (χ^2^(2) = 1.99, *p* = 0.37).

#### Positive predictive value

The PPV after arbitration was 22.1% (3355/15,149; 95% CI: 21.5%, 22.8%) for blinded and 20.6% (6301/30,619; 95% CI: 20.1%, 21.0%) when not blinded (χ^2^(1) = 14.9, *p* < 0.001) (Table 4).

**Table 4:** Two by two table of positive predictive value (PPV) for both after arbitration and for reader 2 against whether reader 2 is blinded or not. Chi-squared test for independence assesses whether PPV after arbitration is independent of blinding (χ^2^(1) = 14.9, *p* < 0.001) and whether PPV of reader 2 is independent of blinding (χ^2^(1) = 28.7, *p* < 0.001)

	Reader 2 Blinded	Reader 2 Not blinded	Total
Cancer detected (TP) after arbitration	3355	6301	9656
Recall (after arbitration), no cancer detected (FP)	11,794	24,318	36,112
Total recalled (TP + FP)	15,149	30,619	45,768
PPV (TP/total recalled) %	22.1%	20.6%	**-**
Cancer detected (TP) (reader 2)	3226	6117	9343
Recall (reader 2), no cancer detected (FP)	14,877	32,072	46,949
Total recalled (TP + FP)	18,103	38,189	56,292
PPV (TP/total recalled)%	17.8%	16.0%	**-**

#### Further test accuracy estimates based on interval cancer data

Interval cancers within 3 years of screening were used to estimate test accuracy metrics and are shown in Table 5. The estimated sensitivity was 82.44% (3399/4123; 95% CI: 81.28%, 83.60%) for blinded and 82.60% (6391/7737; 95% CI: 81.76%, 83.45%) for not blinded (χ^2^(1) = 0.04, *p* = 0.84). The difference for specificity was statistically significant: 96.89% for blinded and 96.68% for not blinded (χ^2^(1) = 37.6, *p* < 0.001).

**Table 5:** Test accuracy statistic estimates for reader 2 blinded or not blinded. Cancer includes those detected at the screen and interval cancers within 3 years of the screen

		Reader 2 Blinded				Reader 2 Not blinded				Equality of proportions tests	
		Cancer				Cancer					
		Detected	Interval	None	Total	Detected	Interval	None	Total		
Recall (after arbitration)	Yes	3355	44	11,750	15,149	6301	90	24,228	30,619	**-**	
	No	0	724	366,617	367,341	0	1346	704,736	706,082	**-**	
	Total	3355	768	378,367	382,490	6301	1436	728,964	736,701	**-**	
Test accuracy statistics				95% confidence interval				95% confidence interval		χ^2^(1)	*p*
Sensitivity		82.44%		(81.28%,83.60%)		82.60%		(81.76%,83.45%)		0.0390	0.843
Specificity		96.89%		(96.84%,96.95%)		96.68%		(96.64%,96.72%)		37.6	< 0.001
PPV		22.44%		(21.77%,23.10%)		20.87%		(20.42%,21.33%)		14.7	< 0.001
NPV		99.80%		(99.79%,99.82%)		99.81%		(99.80%,99.82%)		0.491	0.483

*PPV* positive predictive value, *NPV* negative predictive value

Number of women with cancer used in the “test accuracy” statistics found by adding the detected and interval cancers together

## Discussion

### Summary of results

We examined the effect that blinding reader 2 to the decision of reader 1 had on behaviour and outcomes using data from the English Breast Cancer Screening Programme. When reader 1 recalled, the probability of reader 2 recalling was around 5% points lower when blinded versus not (69.8% vs 74.7%), suggesting that without blinding they are influenced by the decision of reader 1 and alliterative bias is present. This has the potential to increase recall rates by bypassing arbitration in systems where there is arbitration of discordant decisions. We found that the overall odds of recalling women for further tests were lower and specificity was higher when reader 2 was blinded to the decision of reader 1 compared to when not blinded. Similarly, the PPV after arbitration when reader 2 was blinded was slightly higher (22.1%) versus when not (20.6%, *p* < 0.001). We also found a difference (albeit smaller) in reader 1 recall rates when reader 2 was blinded versus unblinded. This may be due to reader 1 changing their behaviour in anticipation of reader 2 viewing their decision, a training effect from independent reading, or it may be an indication of centre level confounding.

### Comparison with the literature

We identified only one study that directly statistically compared the effects of blinding reader 2 compared to not blinding reader 2 in the setting of a breast cancer screening programme [11]. This study used a system of recalling all discordant results. Klompenhouwer et al [11] found that when reader 2 was not informed of the decision of reader 1, the sensitivity of the screening programme was higher (83.1% vs 75.5%), recall rate was higher (3.3% vs 2.9%), false positive referrals were higher (2.6% vs 2.2%), and the interval cancer rate was lower (1.5 per 1000 screens vs 2.1 per 1000 screens). There was no difference in PPV, cancer detection rate, or proportion of BI-RADS 4 or 5. This provides some evidence of the impact of blinding, but is not applicable to screening programmes where discordant decisions are arbitrated.

Follow on studies assessed the impact of arbitration versus no arbitration of discrepant readings for both blinded and non-blinded reading [13, 14]. To do this, they randomly assigned a third reader to decide retrospectively whether to recall a discrepant reading [13]. Although blinded double reading with arbitration was not directly statistically compared to non-blinded double reading with arbitration, the recall rate was lower for blinded reading 2.2% versus 2.3% for non-blinded reading, PPV was higher 31.2% compared to 27.5%, and cancer detection rate was 6.8 per 1000 screens versus 6.3 per 1000 screens with the proportion of BI-RADS 0 (low suspicion lesions) among recalls at 23.0% versus 26.7%. Sensitivity was 76% for blinded versus 72.7%. Our results show this effect of increased PPV and decreased recall rate with blinding is present also in clinical practice, and is statistically significant. Both studies are inconclusive on the effect of blinding on cancer detection and sensitivity, with trends towards increases which are not statistically significant.

In summary, the previous studies in the Dutch screening programme have shown that when all discordant decisions are recalled, blinding increases cancers detected at screening, and number of false positive recalls to assessment, but with similar PPV. They projected that in screening programmes with arbitration blinding may increase PPV; this was a retrospective analysis rather than prospective measurement. Our study findings aligns with these and expands them. In clinical practice where arbitration is used, our study suggests that blinding improves PPV through increases to specificity. We also found evidence of alliterative bias, which explains the mechanism of action of these effects.

### Strengths and limitations

This study has a number of key strengths. For example, we used a large dataset that was collected as part of a breast screening programme, which included a representative sample of screening centres and women in England, and had very little missing data. We also used a Bayesian approach to modelling, fitting models with MCMC methods. These methods generate a sample from the posterior probability distribution of the parameter which can then be summarised by giving the probability of the coefficient being greater/smaller than 0. This enabled us to assess whether the evidence was compelling enough that the cancer detection rate may increase when the second reader is blinded. Overreliance on the use of a statistically significant cut-off level under frequentist inference may lead to the dismissal of clinically relevant effects [26–28]. Our research provided Bayesian *p* values as an additional measure which can convey the strength of the blinding effect.

The study has limitations. Our data are observational, so we cannot conclude that blinding is causing the improvement in PPV and reduction in recall. Reader 2 was also shown to perform better than reader 1 under both blinded and non-blinded conditions, suggesting that potentially more experienced and senior readers read second more frequently. To reduce this potential bias, trainee readers were removed from the population sample. In this study, we measured readers’ decisions and the woman’s outcomes, but no measurements were made of reading behaviour or how the second reader may have used the first reader’s decision. The blinded versus non-blinded improvement is also seen to a lesser extent in reader 1 which cannot be caused by the alliterative effect. Services that used blinding could have more experienced readers overall or could serve a different population demographic of screened women (e.g. by ethnicity, socioeconomic status). Differences between centres were however addressed by clustering by centre and reader as well as controlling by age and screening status. Finally, our 5% rule for designating a centre as blinded/not blinded/mixed was arbitrarily selected.

### Policy implications

In breast screening programmes with arbitration of discordant decisions between readers, blinding the second reader to the decision of the first may improve the PPV of breast cancer screening and reduce the number of women recalled for further testing. The results suggest that reader 2 might be influenced by, and conform to, reader 1’s decisions when not blinded, particularly if a woman has been recalled by reader 1 (potential alliterative bias). So when reader 2 is not blinded, they appear to be copying some of the recall decisions of reader 1, and therefore bypassing the arbitration process and increasing recall rates and false positives. A previous study (where the arbitration was in a laboratory rather than screening practice context) predicted similar patterns [13, 14]. The effect on cancer detection rate is unclear, but the point estimates were higher when blinded in both studies. These results are not generalizable to screening programmes where all discordant decisions are recalled. In that context, previous research has suggested blinding increases cancer detection and false positive recall, whilst maintaining similar PPV.

## Conclusions

Our results suggest that when not blinded reader 2 is influenced by reader 1’s decisions to recall (alliterative bias) which would result in bypassing arbitration and negate some of the benefits of double reading. We found a relationship between blinding the second reader and slightly higher PPV of breast cancer screening, although this analysis may be confounded by other centre characteristics. We would recommend blinded over non-blinded double reading in centres that use arbitration of discordant decisions.

## Supplementary Information

ESM 1 (DOCX 1934 kb)

## Abbreviations

DCIS: Ductal carcinoma in situ
DIC: Deviance information criterion
ESS: Effective sample size
MCMC: Markov chain Monte Carlo
MLwiN: Statistical Software Package for Fitting Multilevel Models
NBSS: National Breast Screening System
NPV: Negative predictive value
PPV: Positive predictive value
STROBE: Strengthening the Reporting of Observational Studies in Epidemiology

## Declarations

### Guarantor

The scientific guarantor of this publication is Professor Sian Taylor-Phillips.

### Statistics and biometry

David Jenkinson has significant statistical expertise and is a statistician at the University of Warwick and one of the authors. Jennifer Cooper is a Senior Research Associate in Medical Statistics at the University of Bristol. Professor Sian Taylor-Phillips is a Professor of Population Health for Warwick Screening at Warwick Medical School.

### Study subjects or cohorts overlap

None of the outcomes in the current paper have been previously reported. Some study subjects or cohorts have been previously reported in:

Taylor-Phillips S, Wallis MG, Jenkinson D et al (2016) Effect of Using the Same vs Different Order for Second Readings of Screening Mammograms on Rates of Breast Cancer Detection: A Randomized Clinical Trial. JAMA 315:1956-1965

Taylor-Phillips S, Jenkinson D, Stinton C, Wallis MG, Dunn J, Clarke A (2018) Double Reading in Breast Cancer Screening: Cohort Evaluation in the CO-OPS Trial. Radiology 287:749-757

Cooper JA, Jenkinson D, Taylor-Phillips S (2019) Blinding of the second reader in mammography screening: impact on behaviour and cancer detection", Proc. SPIE 10952, Medical Imaging 2019: Image Perception, Observer Performance, and Technology Assessment, 109520T (4 March 2019)

### Methodology

• Observational cohort

• Multicentre study
