# Supplementary material for: Optimising breast cancer screening reading: blinding the second reader to the first reader’s decisions
Source: Eur Radiol. 2021 Jun 12;32(1):602–12. doi: 10.1007/s00330-021-07965-z (PMC8660753; doi:10.1007/s00330-021-07965-z)
Supplement: Supplementary file 1 — (DOCX 1934 kb) [file 330_2021_7965_MOESM1_ESM.docx]

# Electronic Supplementary Material (ESM): APPENDICES

**Model Details**
The models used 100,000 iterations after a 10,000 burn-in period, and one chain. The convergence of the Markov Chain Monte Carlo (MCMC) model was evaluated using visual plots (trace, autocorrelation and kernel density plots). An MCMC approach can obtain model estimates for more complex models where standard estimation using maximum likelihood estimation may breakdown [1; 2]. A Bayesian approach based upon MCMC methods generates a sample from the posterior probability distribution of the parameter which can then be summarised by giving the probability of the coefficient being greater/smaller than zero. This enables us to assess whether the evidence is compelling enough to suggest whether an outcome has a higher probability of occurrence for a particular intervention without overreliance on a particular p-value under frequentist inference which may lead to dismissal of a potentially clinical effect [3-5]. This is particularly relevant in today’s context where the use of significance values as thresholds is increasingly debated. It has therefore been suggested that p values and confidence intervals are supplemented with an additional measure which can convey the strength of effect [5]. The approach used in this study provides the Bayesian p-value to assess the strength of an association for decision making and future research.

**References**:

1 Hamra G, MacLehose R, Richardson D (2013) Markov chain Monte Carlo: an introduction for epidemiologists. Int J Epidemiol 42:627-634

2 Goldstein H, Browne W, Rasbash J (2002) Multilevel modelling of medical data. Stat Med 21:3291-3315

3 Amrhein V, Greenland S, McShane B (2019) Scientists rise up against statistical significance. Nature 567:305-307

4 Wasserstein RL, Schirm AL, Lazar NA (2019) Moving to a World Beyond “p < 0.05”. The American Statistician 73:1-19

5 Colquhoun D (2019) The False Positive Risk: A Proposal Concerning What to Do About p-Values. The American Statistician 73:192-201

## Appendix A: Recall Rate and Cancers Detected Models (Overall and by Reader 2)

| **Fixed Effects** | **Coefficient^a^** | **Standard Error** | **z** | **Pr(>\|z\|)^b^** | **95% Credible Interval for the coefficient^c^** | **ESS^d^** | **Odds Ratio^e^** | **95% Credible Interval for the Odds Ratio^f^** | **Bayesian p value*^g^*** |
| --- | --- | --- | --- | --- | --- | --- | --- | --- | --- |
| **Intercept** | -3.444 | 0.038 | -89.66 | <0.001 | -3.522, -3.369 | 186 | - | - | - |
| **Blinding Yes (versus no as the reference category)** | -0.141 | 0.026 | -5.52 | <0.001 | -0.191, -0.091 | 1,411 | 0.869 | 0.826, 0.913 | 0.000 |
| **Age (centred)** | 0.007 | 0.001 | 6.37 | <0.001 | 0.005, 0.009 | 2,655 | 1.007 | 1.005, 1.009 | 1.000 |
| **First Screen (versus Subsequent Screen as the reference category)** | 1.038 | 0.017 | 59.4 | <0.001 | 1.004, 1.072 | 2,319 | 2.823 | 2.728, 2.922 | 1.000 |
| **Blinded Yes * Age (interaction term)** | 0.005 | 0.002 | 2.43 | 0.015 | 0.001, 0.008 | 2,763 | 1.005 | 1.001, 1.008 | 0.992 |
| **Blinding Yes * First Screen** | 0.057 | 0.03 | 1.9 | 0.057 | -0.001, 0.118 | 2,388 | 1.060 | 0.999, 1.125 | 0.974 |
| **Random Effects** | | | | | | | | | |
| Intercept Centre Level 3 | 0.058 | 0.014 | - | - | 0.037, 0.091 | 21,456 | - | - | - |
| Intercept Reader Level 2 | 0.015 | 0.014 | - | - | 0.001, 0.039 | 1 | - | - | - |
| ***^a^****The mean of the 100,000 chain iterations produced by the MCMC multilevel logistic regression model.* ***^b^****Two tailed p value of the z score for the coefficient (testing whether the estimate is significantly different from zero assuming normality)* ***^c^****95% credible interval generated by taking the 2.5th and 97.5th quantiles of the 100,000 chain iterations produced by the MCMC model* ***^d^****Effective Sample Size (ESS) the number of iterations required to obtain the estimate (independent, identically distributed sample)* **^e^**The mean of the 100,000 chain iterations after converting from the log odds scale to the odds scale. ***^f^****95% credible interval is generated by taking the 2.5th and 97.5th quantiles of the 100,000 chain iterations after converting from the log odds scale to the odds scale.* ***^g^****Bayesian p value: The proportion of the 100,000 chain iterations that were above zero (i.e. odds ratios above one). This provides an estimate of the posterior probability that the odds ratio is above one; although care needs to be taken when all iterations are on the same side, leading to a Bayesian p value of 0 or 1.* | | | | | | | | | |

**Table A.1:** Markov Chain Monte Carlo (MCMC) multilevel model determining the effect of blinding on recall rate overall (after arbitration). Interactions retained were blinded by age and blinded by a first screen. The one-sided Bayesian p values give the proportion of chain iterations which are above zero (i.e. odds ratios above one).

| **Age** | **Screen Status (First Screen versus Subsequent Screen)** | **Log Odds Ratio (of blinded to not blinded)** | | | **Odds Ratio (of blinded to not blinded)** | | |
| --- | --- | --- | --- | --- | --- | --- | --- |
|  |  | **Median** | **95% Credible Interval** | | **Median** | **95% Credible Interval** | |
| 50 | Subsequent Screen | -0.183 | -0.248 | -0.118 | 0.833 | 0.780 | 0.889 |
| 50 | First Screen | -0.125 | -0.181 | -0.070 | 0.882 | 0.834 | 0.933 |
| 55 | Subsequent Screen | -0.160 | -0.215 | -0.106 | 0.852 | 0.806 | 0.900 |
| 55 | First Screen | -0.103 | -0.161 | -0.044 | 0.902 | 0.851 | 0.957 |
| 60 | Subsequent Screen | -0.138 | -0.187 | -0.088 | 0.871 | 0.829 | 0.915 |
| 60 | First Screen | -0.081 | -0.146 | -0.014 | 0.923 | 0.864 | 0.986 |
| 65 | Subsequent Screen | -0.115 | -0.166 | -0.065 | 0.891 | 0.847 | 0.938 |
| 65 | First Screen | -0.058 | -0.135 | 0.020 | 0.944 | 0.874 | 1.021 |
| 70 | Subsequent Screen | -0.093 | -0.151 | -0.035 | 0.911 | 0.860 | 0.966 |
| 70 | First Screen | -0.036 | -0.126 | 0.056 | 0.965 | 0.882 | 1.058 |

**Table A.2**: Log odds ratios and odds ratios (of blinded to not blinded) for recall rate overall at different ages and by first screen/subsequent screen. Odds ratios are the median plus the 95% credible intervals.

| **Fixed Effects** | **Coefficient^a^** | **Standard Error** | **z** | **Pr(>\|z\|)^b^** | **95% Credible Interval for the coefficient^c^** | **ESS^d^** | **Odds Ratio^e^** | **95% Credible Interval for the Odds Ratio^f^** | **Bayesian p value*^g^*** |
| --- | --- | --- | --- | --- | --- | --- | --- | --- | --- |
| **Intercept** | -3.264 | 0.048 | -67.36 | <0.001 | -3.360, -3.170 | 103 | - | - | - |
| **Blinding Yes (versus no as the reference category)** | -0.162 | 0.026 | -6.22 | <0.001 | -0.213,-0.111 | 1,150 | 0.851 | 0.808, 0.895 | 0.000 |
| **Age (centred)** | 0.004 | 0.001 | 4.63 | <0.001 | 0.003, 0.006 | 3,254 | 1.004 | 1.003, 1.006 | 1.000 |
| **First Screen (versus Subsequent Screen as the reference category)** | 0.99 | 0.016 | 63.65 | <0.001 | 0.959, 1.020 | 2,546 | 2.691 | 2.610, 2.773 | 1.000 |
| **Blinded Yes * Age (interaction term)** | 0.004 | 0.002 | 2.19 | 0.028 | 0.0004, 0.0070 | 3,252 | 1.004 | 1.0004, 1.0071 | 0.986 |
| **Blinding Yes * First Screen** | 0.047 | 0.028 | 1.72 | 0.086 | -0.007, 0.102 | 2,399 | 1.049 | 0.993, 1.107 | 0.958 |
| **Random Effects** | | | | | | | | | |
| Intercept Centre Level 3 | 0.089 | 0.021 | - | - | 0.057, 0.139 | 9,734 | - | - | - |
| Intercept Reader Level 2 | 0.074 | 0.005 | - | - | 0.064, 0.084 | 111 | - | - | - |
| ***^a^****The mean of the 100,000 chain iterations produced by the MCMC multilevel logistic regression model.* ***^b^****Two tailed p value of the z score for the coefficient (testing whether the estimate is significantly different from zero assuming normality)* ***^c^****95% credible interval generated by taking the 2.5th and 97.5th quantiles of the 100,000 chain iterations produced by the MCMC model* ***^d^****Effective Sample Size (ESS) the number of iterations required to obtain the estimate (independent, identically distributed sample)* **^e^**The mean of the 100,000 chain iterations after converting from the log odds scale to the odds scale. ***^f^****95% credible interval is generated by taking the 2.5th and 97.5th quantiles of the 100,000 chain iterations after converting from the log odds scale to the odds scale.* ***^g^****Bayesian p value: The proportion of the 100,000 chain iterations that were above zero (i.e. odds ratios above one). This provides an estimate of the posterior probability that the odds ratio is above one; although care needs to be taken when all iterations are on the same side, leading to a Bayesian p value of 0 or 1.* | | | | | | | | | |

**Table A.3:** Markov Chain Monte Carlo (MCMC) multilevel model determining the effect of blinding on reader 2 recall rate (without arbitration). Interactions retained were blinded by age and blinded by a first screen. The one-sided Bayesian p values give the proportion of chain iterations which are above zero (i.e. odds ratios above one).

| **Age** | **Screen Status (First Screen versus Subsequent Screen)** | **Log Odds Ratio (of blinded to not blinded)** | | | **Odds Ratio (of blinded to not blinded)** | | |
| --- | --- | --- | --- | --- | --- | --- | --- |
|  |  | **Median** | **95% Credible Interval** | | **Median** | **95% Credible Interval** | |
| 50 | Subsequent Screen | -0.196 | -0.259 | -0.132 | 0.822 | 0.772 | 0.876 |
| 50 | First Screen | -0.148 | -0.204 | -0.094 | 0.862 | 0.815 | 0.911 |
| 55 | Subsequent Screen | -0.177 | -0.233 | -0.123 | 0.837 | 0.792 | 0.885 |
| 55 | First Screen | -0.130 | -0.188 | -0.073 | 0.878 | 0.829 | 0.929 |
| 60 | Subsequent Screen | -0.159 | -0.210 | -0.108 | 0.853 | 0.810 | 0.897 |
| 60 | First Screen | -0.111 | -0.175 | -0.048 | 0.895 | 0.839 | 0.953 |
| 65 | Subsequent Screen | -0.140 | -0.192 | -0.090 | 0.869 | 0.825 | 0.914 |
| 65 | First Screen | -0.093 | -0.167 | -0.020 | 0.911 | 0.846 | 0.980 |
| 70 | Subsequent Screen | -0.122 | -0.179 | -0.065 | 0.885 | 0.836 | 0.937 |
| 70 | First Screen | -0.075 | -0.160 | 0.010 | 0.928 | 0.852 | 1.010 |

**Table A.4:** Log odds ratios and odds ratios (of blinded to not blinded) for recall rate by reader 2 without arbitration at different ages and by first screen/subsequent screen. Odds ratios are the median plus the 95% credible intervals.


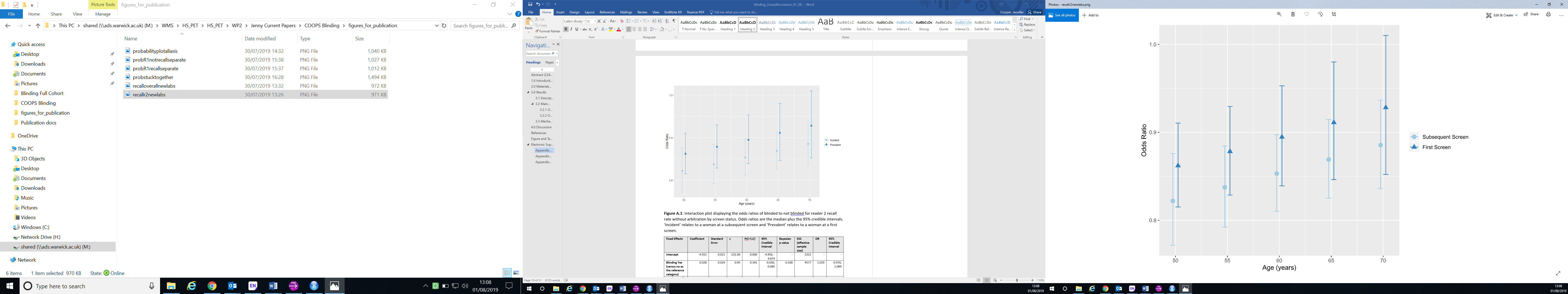


**Figure A.1**: Interaction plot displaying the odds ratios of blinded to not blinded for reader 2 recall rate without arbitration by screen status. Odds ratios are the median plus the 95% credible intervals.

| **Fixed Effects** | **Coefficient^a^** | **Standard Error** | **z** | **Pr(>\|z\|)^b^** | **95% Credible Interval for the coefficient^c^** | **ESS^d^** | **Odds Ratio^e^** | **95% Credible Interval for the Odds Ratio^f^** | **Bayesian p value*^g^*** |
| --- | --- | --- | --- | --- | --- | --- | --- | --- | --- |
| **Intercept** | -4.915 | 0.021 | -232.60 | 0.000 | -4.956, -4.874 | 3,323 | - | - | - |
| **Blinding Yes (versus no as the reference category)** | 0.028 | 0.029 | 0.95 | 0.341 | -0.030, 0.085 | 4,577 | 1.029 | 0.970, 1.089 | 0.832 |
| **Age (centred)** | 0.051 | 0.002 | 31.66 | <0.001 | 0.048, 0.054 | 7,324 | 1.052 | 1.049, 1.056 | 1.000 |
| **First Screen (versus Subsequent Screen as the reference category)** | 0.528 | 0.033 | 16.23 | <0.001 | 0.464, 0.591 | 6,910 | 1.696 | 1.591, 1.807 | 1.000 |
| **Random Effects** | | | | | | | | | |
| Intercept Centre Level 3 | 0.006 | 0.003 | - | - | 0.002, 0.011 | 5,367 | - | - | - |
| Intercept Reader Level 2 | 0.001 | 0.001 | - | - | 0.0002, 0.003 | 2 | - | - | - |
| ***^a^****The mean of the 100,000 chain iterations produced by the MCMC multilevel logistic regression model.* ***^b^****Two tailed p value of the z score for the coefficient (testing whether the estimate is significantly different from zero assuming normality)* ***^c^****95% credible interval generated by taking the 2.5th and 97.5th quantiles of the 100,000 chain iterations produced by the MCMC model* ***^d^****Effective Sample Size (ESS) the number of iterations required to obtain the estimate (independent, identically distributed sample)* **^e^**The mean of the 100,000 chain iterations after converting from the log odds scale to the odds scale. ***^f^****95% credible interval is generated by taking the 2.5th and 97.5th quantiles of the 100,000 chain iterations after converting from the log odds scale to the odds scale.* ***^g^****Bayesian p value: The proportion of the 100,000 chain iterations that were above zero (i.e. odds ratios above one). This provides an estimate of the posterior probability that the odds ratio is above one; although care needs to be taken when all iterations are on the same side, leading to a Bayesian p value of 0 or 1.* | | | | | | | | | |

**Table A.5:** Markov Chain Monte Carlo (MCMC) multilevel model determining the effect of blinding on cancer detection overall (with arbitration) adjusted for age and whether the woman is at her first screen or subsequent screen. The one-sided Bayesian p values give the proportion of chain iterations which are above zero (i.e. odds ratios above one).

| **Fixed Effects** | **Coefficient^a^** | **Standard Error** | **z** | **Pr(>\|z\|)^b^** | **95% Credible Interval for the coefficient^c^** | **ESS^d^** | **Odds Ratio^e^** | **95% Credible Interval for the Odds Ratio^f^** | **Bayesian p value*^g^*** |
| --- | --- | --- | --- | --- | --- | --- | --- | --- | --- |
| **Intercept** | -4.945 | 0.021 | -237.49 | <0.001 | -4.987, -4.904 | 3,380 | - | - | - |
| **Blinding Yes (versus no as the reference category)** | 0.021 | 0.029 | 0.73 | 0.466 | -0.036, 0.079 | 4,587 | 1.022 | 0.964, 1.082 | 0.768 |
| **Age (centred)** | 0.051 | 0.002 | 30.80 | <0.001 | 0.048, 0.054 | 7,437 | 1.052 | 1.049, 1.055 | 1.000 |
| **First Screen (versus Subsequent Screen as the reference category)** | 0.527 | 0.033 | 15.82 | <0.001 | 0.461, 0.593 | 6,734 | 1.695 | 1.586, 1.809 | 1.000 |
| **Random Effects** | | | | | | | | | |
| Intercept Centre Level 3 | 0.005 | 0.003 | - | - | 0.002, 0.011 | 4,566 | - | - | - |
| Intercept Reader Level 2 | 0.001 | 0.001 | - | - | 0.0001, 0.003 | 4 | - | - | - |
| ***^a^****The mean of the 100,000 chain iterations produced by the MCMC multilevel logistic regression model.* ***^b^****Two tailed p value of the z score for the coefficient (testing whether the estimate is significantly different from zero assuming normality)* ***^c^****95% credible interval generated by taking the 2.5th and 97.5th quantiles of the 100,000 chain iterations produced by the MCMC model* ***^d^****Effective Sample Size (ESS) the number of iterations required to obtain the estimate (independent, identically distributed sample)* **^e^**The mean of the 100,000 chain iterations after converting from the log odds scale to the odds scale. ***^f^****95% credible interval is generated by taking the 2.5th and 97.5th quantiles of the 100,000 chain iterations after converting from the log odds scale to the odds scale.* ***^g^****Bayesian p value: The proportion of the 100,000 chain iterations that were above zero (i.e. odds ratios above one). This provides an estimate of the posterior probability that the odds ratio is above one; although care needs to be taken when all iterations are on the same side, leading to a Bayesian p value of 0 or 1.* | | | | | | | | | |

**Table A.6:** MCMC multilevel model determining the effect of blinding on cancer detection by Reader 2 (without arbitration) adjusted for age and whether the woman is at her first screen or subsequent screen. The one-sided Bayesian p values give the proportion of chain iterations which are above zero (i.e. odds ratios above one).


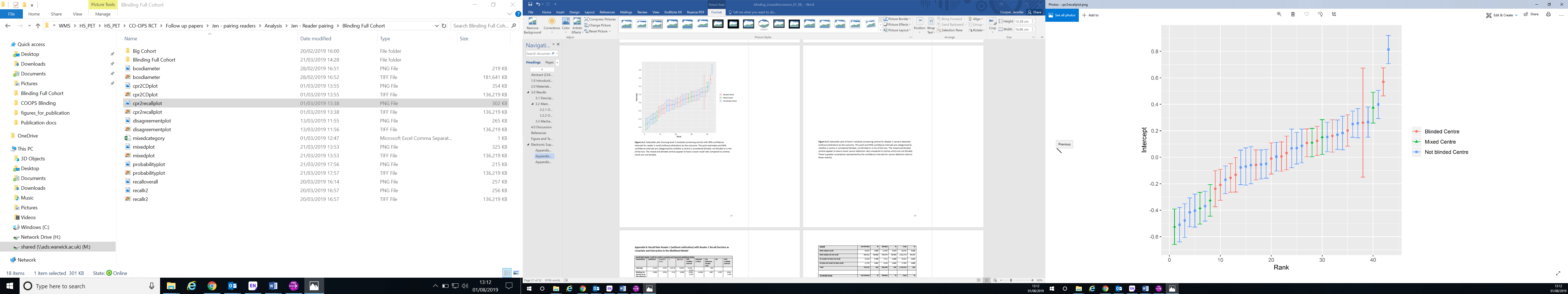


**Figure A.2:** Caterpillar plot showing level 3 residuals (screening centre) with 95% confidence intervals for reader 2 recall (without arbitration) as the outcome. The point estimates and 95% confidence intervals are categorised by whether a centre is considered blinded, not blinded or a mix of the two. The mixed and blinded centres appear to have a lower recall rate compared to centres which are not blinded.


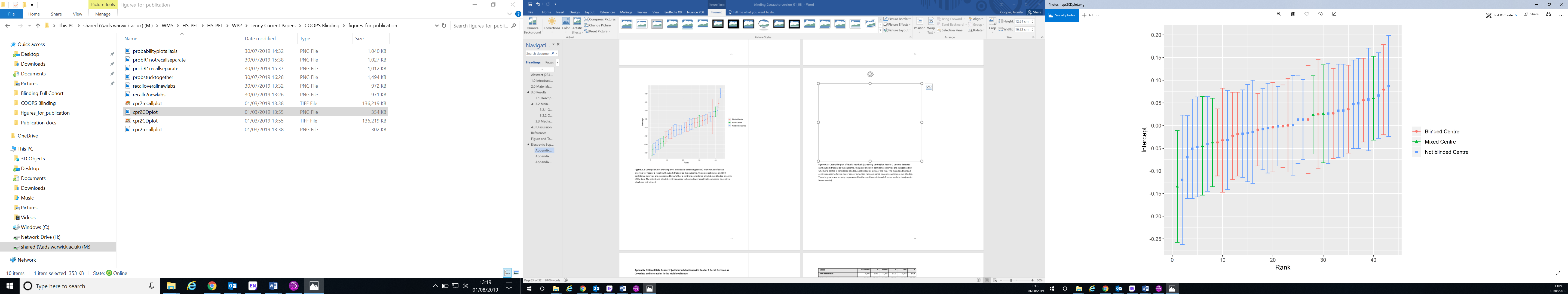


**Figure A.3:** Caterpillar plot of level 3 residuals (screening centre) for Reader 2 cancers detected (without arbitration) as the outcome. The point and 95% confidence intervals are categorised by whether a centre is considered blinded, not blinded or a mix of the two. The mixed and blinded centres appear to have a lower cancer detection rate compared to centres which are not blinded. There is greater uncertainty represented by the confidence intervals for cancer detection (due to fewer events).

## Appendix B: Recall Rate Reader 2 (without arbitration) with Reader 1 Recall Decision as Covariate and Interaction in the Multilevel Model

| **Fixed Effects** | **Coefficient^a^** | **Standard Error** | **z** | **Pr(>\|z\|)^b^** | **95% Credible Interval for the coefficient^c^** | **ESS^d^** | **Odds Ratio^e^** | **95% Credible Interval for the Odds Ratio^f^** | **Bayesian p value*^g^*** |
| --- | --- | --- | --- | --- | --- | --- | --- | --- | --- |
| **Intercept** | -4.348 | 0.037 | -116.19 | <0.001 | -4.419, -4.271 | 306 | - | - | - |
| **Blinding Yes (versus no as the reference category)** | 0.006 | 0.034 | 0.18 | 0.858 | -0.061, 0.074 | 1,097 | 1.007 | 0.941, 1.076 | 0.572 |
| **Reader 1 recall (not recall as reference category)** | 4.825 | 0.016 | 305.04 | <0.001 | 4.794, 4.856 | 2,748 | 124.606 | 120.745, 128.505 | 1.000 |
| **Age (centred)** | 0.005 | 0.001 | 4.45 | <0.001 | 0.003, 0.007 | 6,372 | 1.005 | 1.003, 1.007 | 0.99995 |
| **First Screen (versus Subsequent Screen as the reference category)** | 0.604 | 0.017 | 34.61 | <0.001 | 0.570, 0.639 | 5,215 | 1.830 | 1.768, 1.894 | 1.000 |
| **Blinded Yes * Reader 1 recall (interaction term)** | -0.251 | 0.025 | -9.93 | <0.001 | -0.300, -0.201 | 5,000 | 0.778 | 0.741, 0.818 | 0.000 |
| **Random Effects** | | | | | | | | | |
| Intercept Centre Level 3 | 0.049 | 0.012 | - | - | 0.031, 0.077 | 13,716 | - | - | - |
| Intercept Reader Level 2 | 0.230 | 0.010 | - | - | 0.210, 0.250 | 430 | - | - | - |
| ***^a^****The mean of the 100,000 chain iterations produced by the MCMC multilevel logistic regression model.* ***^b^****Two tailed p value of the z score for the coefficient (testing whether the estimate is significantly different from zero assuming normality)* ***^c^****95% credible interval generated by taking the 2.5th and 97.5th quantiles of the 100,000 chain iterations produced by the MCMC model* ***^d^****Effective Sample Size (ESS) the number of iterations required to obtain the estimate (independent, identically distributed sample)* **^e^**The mean of the 100,000 chain iterations after converting from the log odds scale to the odds scale. ***^f^****95% credible interval is generated by taking the 2.5th and 97.5th quantiles of the 100,000 chain iterations after converting from the log odds scale to the odds scale.* ***^g^****Bayesian p value: The proportion of the 100,000 chain iterations that were above zero (i.e. odds ratios above one). This provides an estimate of the posterior probability that the odds ratio is above one; although care needs to be taken when all iterations are on the same side, leading to a Bayesian p value of 0 or 1.* | | | | | | | | | |

**Table B.1:**  Markov Chain Monte Carlo (MCMC) multilevel model determining the effect of blinding and whether reader 1 recalls or not on reader 2 recall rate (without arbitration). Reader 1 recall by whether reader 2 is blinded is included as an interaction term. The one-sided Bayesian p values give the proportion of chain iterations which are above zero (i.e. odds ratios above one).

| **Overall** | **Not blinded** | **%** | **Blinded** | **%** | **Total** | **%** |
| --- | --- | --- | --- | --- | --- | --- |
| **Both readers recall** | 26,397 | **3.583** | 11,945 | **3.123** | 38,342 | **3.426** |
| **Both readers do not recall** | 685,502 | **93.050** | 356,876 | **93.303** | 1,042,378 | **93.137** |
| **R1 recalls, R2 does not recall** | 13,010 | **1.766** | 7,511 | **1.964** | 20,521 | **1.834** |
| **R1 does not recall, R2 does recall** | 11,792 | **1.601** | 6,158 | **1.610** | 17,950 | **1.604** |
| **Total** | **736,701** | **100** | **382,490** | **100** | **1,119,191** | **100** |
|  |  |  |  |  |  |  |
| **R1 Recalls Group** | **Not blinded** | **%** | **Blinded** | **%** | **Total** | **%** |
| **R1 Recall - Agreement (both recall)** | 26,397 | **66.986** | 11,945 | **61.395** | 38,342 | **65.138** |
| **R1 recall, R2 does not (disagreement)** | 13,010 | **33.014** | 7,511 | **38.605** | 20,521 | **34.862** |
| **Total (for R1 recalls group)** | 39,407 | **100** | 19,456 | **100** | 58,863 | **100** |
|  |  |  |  |  |  |  |
| **R1 Does Not Recall Group** | **Not blinded** | **%** | **Blinded** | **%** | **Total** | **%** |
| **R1 does not recall – Agreement (both do not recall)** | 685,502 | **98.309** | 356,876 | **98.304** | 1,042,378 | **98.307** |
| **R1 does not recall, R2 does (disagreement)** | 11,792 | **1.691** | 6,158 | **1.696** | 17,950 | **1.693** |
| **Total (for R1 does not recall group)** | **697,294** | **100** | **363,034** | **100** | **1,060,328** | **100** |

**Table B.2:** Disagreement/agreement rates between reader 1 and reader 2 when reader 2 is blinded and not blinded. These data are also displayed by the subgroups of whether reader 1 recalls, or whether reader 1 does not recall. R1= Reader 1, R2 = Reader 2.

| **Scenario** | | | **Mean probability  of R2 recall (%)** | **2.50%** | **97.50%** |
| --- | --- | --- | --- | --- | --- |
| **First or Subsequent Screen** | **R1 recalls or R1 does not recall** | **R2 blinded or R2 not blinded** |  |  |  |
| Subsequent Screen | R1 does not recall | R2 not blinded | 1.278 | 1.191 | 1.378 |
| Subsequent Screen | R1 does not recall | R2 blinded | 1.286 | 1.185 | 1.393 |
| Subsequent Screen | R1 does recall | R2 not blinded | 61.712 | 60.004 | 63.483 |
| Subsequent Screen | R1 does recall | R2 blinded | 55.785 | 53.739 | 57.819 |
| First Screen | R1 does not recall | R2 not blinded | 2.315 | 2.151 | 2.497 |
| First Screen | R1 does not recall | R2 blinded | 2.329 | 2.140 | 2.528 |
| First Screen | R1 does recall | R2 not blinded | 74.675 | 73.226 | 76.119 |
| First Screen | R1 does recall | R2 blinded | 69.771 | 67.938 | 71.547 |

**Table B.3:** Probabilities that reader 2 (R2) recalls based on whether reader 2 is blinded, the woman’s screen status and whether reader 1 (R1) recalls (for a woman at average age, 59.27). The probabilities are determined using the model reported in **Table B.1** which includes the interaction term of Reader 1 recall by whether Reader 2 is blinded or not.

## Appendix C: Sensitivity Analysis Mixed Models Only

Sensitivity Analysis includes the six centres which have a mix of both blinded and not blinded second readers. If less than 5% of reader 2’s mammograms were blinded out of the total read for that centre, then the centre was classed as ‘not blinded’. A mixed protocol centre was one where there was at least 5% of blinded or not blinded out of the total number of mammograms read at the centre. If the proportion was under 5% then that centre could be classed as truly ‘blinded’ or ‘not blinded’.


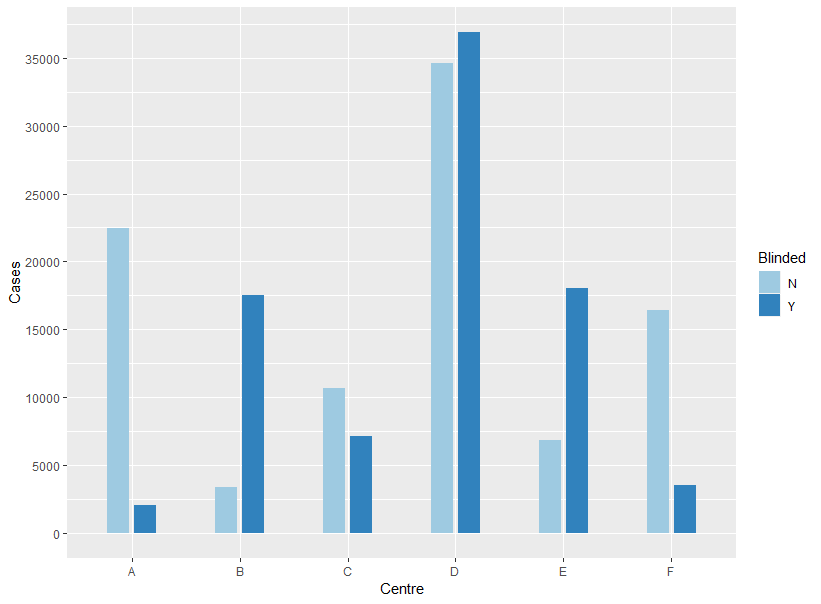


**Figure C.1**: The six mixed screening centres and the proportion of blinded to not blinded women’s mammograms read by reader 2 at that centre.

| **Study Characteristic** |  | **Reader 2 Blinded** | **%** | **Reader 2 Not Blinded** | **%** |
| --- | --- | --- | --- | --- | --- |
| **Age of woman (mean)** | **Mean Age** | **59.6** | - | **59.4** | - |
|  | **Group 1 (52 or less)** | **18,592** | 21.82 | **21,392** | 22.67 |
|  | **Group 2 (53-59)** | **24,538** | 28.80 | **27,399** | 29.03 |
|  | **Group 3 (60 or more)** | **42,074** | 49.38 | **45,578** | 48.30 |
|  | **Total** | **85,204** | 100.00 | **94,369** | 100.00 |
| **First Screen/Subsequent Screen** | **Subsequent Screen** | **70,403** | 82.63 | **77,360** | 81.98 |
|  | **First Screen** | **14,801** | 17.37 | **17,009** | 18.02 |
|  | **Total** | **85,204** | 100.00 | **94,369** | 100.00 |
| **Reader 1 Recall**  **(pre arbitration)** | **Not recalled** | **81,232** | 95.34 | **89,587** | 94.93 |
|  | **Recalled** | **3,972** | 4.66 | **4,782** | 5.07 |
|  | **Total** | **85,204** | 100.00 | **94,369** | 100.00 |
| **Reader 2 Recall (pre arbitration)** | **Not recalled** | **81,415** | 95.55 | **89,506** | 94.85 |
|  | **Recalled** | **3,789** | 4.45 | **4,863** | 5.15 |
|  | **Total** | **85,204** | 100.00 | **94,369** | 100.00 |
| **Recall** | **Not recalled** | **81,796** | 96.00 | **90,352** | 95.74 |
| **(after arbitration)** | **Recalled** | **3,408** | 4.00 | **4,017** | 4.26 |
|  | **Total** | **85,204** | 100.00 | **94,369** | 100.00 |
| **Cancers detected by  Reader 1** | **Cancer detected** | **703** | 0.83 | **720** | 0.76 |
|  | **No Cancer detected** | **84501** | 99.17 | **93649** | 99.24 |
|  | **Total** | **85204** | 100.00 | **94369** | 100.00 |
| **Cancers detected by  Reader 2** | **Cancer detected** | **746** | 0.88 | **760** | 0.81 |
|  | **No Cancer detected** | **84458** | 99.12 | **93609** | 99.19 |
|  | **Total** | **85204** | 100.00 | **94369** | 100.00 |
| **Cancers detected overall (after arbitration)** | **Cancer detected** | **763** | 0.90 | **783** | 0.83 |
|  | **No Cancer detected** | **84,441** | 99.10 | **93,586** | 99.17 |
|  | **Total** | **85,204** | 100.00 | **94,369** | 100.00 |

**Table C.1:** Mixed centres only study population characteristics (n=179,573). Recall and cancer detection rates for blinded versus not blinded reader 2. Reader 2 was blinded for 47.45% of women screened (85,204/179,573).

| **Fixed Effects** | **Coefficient^a^** | **Standard Error** | **z** | **Pr(>\|z\|)^b^** | **95% Credible Interval for the coefficient^c^** | **ESS^d^** | **Odds Ratio^e^** | **95% Credible Interval for the Odds Ratio^f^** | **Bayesian p value*^g^*** |
| --- | --- | --- | --- | --- | --- | --- | --- | --- | --- |
| **Intercept** | -3.471 | 0.164 | -21.12 | <0.001 | -3.900, -3.170 | 60 | - | - | - |
| **Blinding Yes (versus no as the reference category)** | -0.125 | 0.029 | -4.39 | <0.001 | -0.182, -0.070 | 5,977 | 0.883 | 0.834, 0.933 | 0.000 |
| **Age (centred)** | 0.005 | 0.002 | 2.45 | 0.014 | 0.001, 0.009 | 7,013 | 1.005 | 1.001, 1.009 | 0.993 |
| **First Screen (versus Subsequent Screen as the reference category)** | 1.011 | 0.035 | 28.94 | <0.001 | 0.944, 1.081 | 5,584 | 2.752 | 2.570, 2.946 | 1.000 |
| **Random Effects** | | | | | | | | | |
| Intercept Centre Level 3 | 0.143 | 0.184 | - | - | 0.031, 0.556 | 608 | - | - | - |
| Intercept Reader Level 2 | 0.078 | 0.016 | - | - | 0.049, 0.110 | 39 | - | - | - |
| ***^a^****The mean of the 100,000 chain iterations produced by the MCMC multilevel logistic regression model.* ***^b^****Two tailed p value of the z score for the coefficient (testing whether the estimate is significantly different from zero assuming normality)* ***^c^****95% credible interval generated by taking the 2.5th and 97.5th quantiles of the 100,000 chain iterations produced by the MCMC model* ***^d^****Effective Sample Size (ESS) the number of iterations required to obtain the estimate (independent, identically distributed sample)* **^e^**The mean of the 100,000 chain iterations after converting from the log odds scale to the odds scale. ***^f^****95% credible interval is generated by taking the 2.5th and 97.5th quantiles of the 100,000 chain iterations after converting from the log odds scale to the odds scale.* ***^g^****Bayesian p value: The proportion of the 100,000 chain iterations that were above zero (i.e. odds ratios above one). This provides an estimate of the posterior probability that the odds ratio is above one; although care needs to be taken when all iterations are on the same side, leading to a Bayesian p value of 0 or 1.* | | | | | | | | | |

**Table C.2:** Markov Chain Monte Carlo (MCMC) multilevel model for mixed centres only determining the effect of blinding on overall recall rate (after arbitration) adjusted for age, whether the woman is at her first screen or subsequent screen. The one-sided Bayesian p values give the proportion of chain iterations which are above zero (i.e. odds ratios above one).

| **Fixed Effects** | **Coefficient^a^** | **Standard Error** | **z** | **Pr(>\|z\|)^b^** | **95% Credible Interval for the coefficient^c^** | **ESS^d^** | **Odds Ratio^e^** | **95% Credible Interval for the Odds Ratio^f^** | **Bayesian p value*^g^*** |
| --- | --- | --- | --- | --- | --- | --- | --- | --- | --- |
| **Intercept** | -3.274 | 0.121 | -27.05 | <0.001 | -3.530, -3.023 | 103 | - | - | - |
| **Blinding Yes (versus no as the reference category)** | -0.145 | 0.028 | -5.26 | <0.001 | -0.199, -0.091 | 5,587 | 0.865 | 0.819, 0.913 | 0.000 |
| **Age (centred)** | 0.003 | 0.002 | 1.69 | 0.090 | -0.0005, 0.0069 | 7,656 | 1.003 | 0.9995, 1.0069 | 0.553 |
| **First Screen (versus Subsequent Screen as the reference category)** | 0.962 | 0.032 | 29.66 | <0.001 | 0.899, 1.026 | 5,811 | 2.619 | 2.456, 2.789 | 1.000 |
| **Random Effects** | | | | | | | | | |
| Intercept Centre Level 3 | 0.093 | 0.114 | - | - | 0.022, 0.326 | 3,058 | - | - | - |
| Intercept Reader Level 2 | 0.111 | 0.016 | - | - | 0.083, 0.143 | 157 | - | - | - |
| ***^a^****The mean of the 100,000 chain iterations produced by the MCMC multilevel logistic regression model.* ***^b^****Two tailed p value of the z score for the coefficient (testing whether the estimate is significantly different from zero assuming normality)* ***^c^****95% credible interval generated by taking the 2.5th and 97.5th quantiles of the 100,000 chain iterations produced by the MCMC model* ***^d^****Effective Sample Size (ESS) the number of iterations required to obtain the estimate (independent, identically distributed sample)* **^e^**The mean of the 100,000 chain iterations after converting from the log odds scale to the odds scale. ***^f^****95% credible interval is generated by taking the 2.5th and 97.5th quantiles of the 100,000 chain iterations after converting from the log odds scale to the odds scale.* ***^g^****Bayesian p value: The proportion of the 100,000 chain iterations that were above zero (i.e. odds ratios above one). This provides an estimate of the posterior probability that the odds ratio is above one; although care needs to be taken when all iterations are on the same side, leading to a Bayesian p value of 0 or 1.* | | | | | | | | | |

**Table C.3:** Markov Chain Monte Carlo (MCMC) multilevel model for mixed centres only determining the effect of blinding on recall rate by Reader 2 (without arbitration) adjusted for age, whether the woman is at her first screen or subsequent screen and the interaction terms; blinded by age and blinded by a first screen. The one-sided Bayesian p values give the proportion of chain iterations which are above zero (i.e. odds ratios above one).

| **Fixed Effects** | **Coefficient^a^** | **Standard Error** | **z** | **Pr(>\|z\|)^b^** | **95% Credible Interval for the coefficient^c^** | **ESS^d^** | **Odds Ratio^e^** | **95% Credible Interval for the Odds Ratio^f^** | **Bayesian p value*^g^*** |
| --- | --- | --- | --- | --- | --- | --- | --- | --- | --- |
| **Intercept** | -4.927 | 0.054 | -91.22 | <0.001 | -5.034, -4.822 | 2,886 | - | - | - |
| **Blinding Yes (versus no as the reference category)** | 0.074 | 0.054 | 1.36 | 0.1738 | -0.033, 0.180 | 6,014 | 1.078 | 0.968, 1.197 | 0.912 |
| **Age (centred)** | 0.052 | 0.004 | 13.41 | <0.001 | 0.044, 0.059 | 8,508 | 1.053 | 1.045, 1.061 | 1.000 |
| **First Screen (versus Subsequent Screen as the reference category)** | 0.529 | 0.083 | 6.37 | <0.001 | 0.365, 0.692 | 7,865 | 1.704 | 1.441, 1.999 | 1.000 |
| **Random Effects** | | | | | | | | | |
| Intercept Centre Level 3 | 0.005 | 0.010 | - | - | 0.0005, 0.0250 | 4,932 | - | - | - |
| Intercept Reader Level 2 | 0.017 | 0.014 | - | - | 0.0010, 0.0487 | 3 | - | - | - |
| ***^a^****The mean of the 100,000 chain iterations produced by the MCMC multilevel logistic regression model.* ***^b^****Two tailed p value of the z score for the coefficient (testing whether the estimate is significantly different from zero assuming normality)* ***^c^****95% credible interval generated by taking the 2.5th and 97.5th quantiles of the 100,000 chain iterations produced by the MCMC model* ***^d^****Effective Sample Size (ESS) the number of iterations required to obtain the estimate (independent, identically distributed sample)* **^e^**The mean of the 100,000 chain iterations after converting from the log odds scale to the odds scale. ***^f^****95% credible interval is generated by taking the 2.5th and 97.5th quantiles of the 100,000 chain iterations after converting from the log odds scale to the odds scale.* ***^g^****Bayesian p value: The proportion of the 100,000 chain iterations that were above zero (i.e. odds ratios above one). This provides an estimate of the posterior probability that the odds ratio is above one; although care needs to be taken when all iterations are on the same side, leading to a Bayesian p value of 0 or 1.* | | | | | | | | | |

**Table C.4:** Markov Chain Monte Carlo (MCMC) multilevel model for mixed centres only determining the effect of blinding on overall cancer detection rate (with arbitration) adjusted for age, whether the woman is at her first screen or subsequent screen. The one-sided Bayesian p values give the proportion of chain iterations which are above zero (i.e. odds ratios above one).

| **Fixed Effects** | **Coefficient^a^** | **Standard Error** | **z** | **Pr(>\|z\|)^b^** | **95% Credible Interval for the coefficient^c^** | **ESS^d^** | **Odds Ratio^e^** | **95% Credible Interval for the Odds Ratio^f^** | **Bayesian p value*^g^*** |
| --- | --- | --- | --- | --- | --- | --- | --- | --- | --- |
| **Intercept** | -4.953 | 0.052 | -94.68 | <0.001 | -5.054,  -4.849 | 3,451 | - | - | - |
| **Blinding Yes (versus no as the reference category)** | 0.080 | 0.055 | 1.47 | 0.142 | -0.026, 0.188 | 6,523 | 1.085 | 0.974, 1.207 | 0.930 |
| **Age (centred)** | 0.052 | 0.004 | 13.42 | <0.001 | 0.045, 0.060 | 9,256 | 1.053 | 1.046, 1.062 | 1.000 |
| **First Screen (versus Subsequent Screen as the reference category)** | 0.529 | 0.084 | 6.28 | <0.001 | 0.362, 0.694 | 7,914 | 1.703 | 1.437, 2.002 | 1.000 |
| **Random Effects** | | | | | | | | | |
| Intercept Centre Level 3 | 0.005 | 0.012 | - | - | 0.0005, 0.0227 | 18,042 | - | - | - |
| Intercept Reader Level 2 | 0.006 | 0.006 | - | - | 0.0008, 0.0243 | 4 | - | - | - |
| ***^a^****The mean of the 100,000 chain iterations produced by the MCMC multilevel logistic regression model.* ***^b^****Two tailed p value of the z score for the coefficient (testing whether the estimate is significantly different from zero assuming normality)* ***^c^****95% credible interval generated by taking the 2.5th and 97.5th quantiles of the 100,000 chain iterations produced by the MCMC model* ***^d^****Effective Sample Size (ESS) the number of iterations required to obtain the estimate (independent, identically distributed sample)* **^e^**The mean of the 100,000 chain iterations after converting from the log odds scale to the odds scale. ***^f^****95% credible interval is generated by taking the 2.5th and 97.5th quantiles of the 100,000 chain iterations after converting from the log odds scale to the odds scale.* ***^g^****Bayesian p value: The proportion of the 100,000 chain iterations that were above zero (i.e. odds ratios above one). This provides an estimate of the posterior probability that the odds ratio is above one; although care needs to be taken when all iterations are on the same side, leading to a Bayesian p value of 0 or 1.* | | | | | | | | | |

**Table C.5:** Markov Chain Monte Carlo (MCMC) multilevel model for mixed centres only determining the effect of blinding on reader 2 cancer detection rate (without arbitration) adjusted for age, whether the woman is at her first screen or subsequent screen. The one-sided Bayesian p values give the proportion of chain iterations which are above zero (i.e. odds ratios above one).

| **Fixed Effects** | **Coefficient^a^** | **Standard Error** | **z** | **Pr(>\|z\|)^b^** | **95% Credible Interval for the coefficient^c^** | **ESS^d^** | **Odds Ratio^e^** | **95% Credible Interval for the Odds Ratio^f^** | **Bayesian p value*^g^*** |
| --- | --- | --- | --- | --- | --- | --- | --- | --- | --- |
| **Intercept** | -4.398 | 0.147 | -29.89 | <0.001 | -4.685, -4.142 | 126 | - | - | - |
| **Blinding Yes (versus no as the reference category)** | -0.092 | 0.045 | -2.02 | 0.043 | -0.182, -0.004 | 3,265 | 0.913 | 0.834, 0.996 | 0.020 |
| **Reader 1 recall (not recall as reference category)** | 4.950 | 0.047 | 105.66 | <0.001 | 4.859, 5.042 | 2,114 | 141.304 | 128.904, 154.760 | 1.000 |
| **Age (centred)** | 0.003 | 0.002 | 1.35 | 0.176 | -0.002, 0.008 | 7,921 | 1.003 | 0.998, 1.008 | 0.913 |
| **First Screen (versus Subsequent Screen as the reference category)** | 0.627 | 0.045 | 13.82 | <0.001 | 0.539, 0.715 | 6,167 | 1.874 | 1.714, 2.045 | 1.000 |
| **Blinded Yes * Reader 1 recall (interaction term)** | 0.023 | 0.063 | 0.36 | 0.7186 | -0.102, 0.147 | 3,460 | 1.025 | 0.903, 1.159 | 0.639 |
| **Random Effects** | | | | | | | | | |
| Intercept Centre Level 3 | 0.146 | 0.171 | - | - | 0.034, 0.534 | 937 | - | - | - |
| Intercept Reader Level 2 | 0.303 | 0.033 | - | - | 0.241, 0.369 | 297 | - | - | - |
| ***^a^****The mean of the 100,000 chain iterations produced by the MCMC multilevel logistic regression model.* ***^b^****Two tailed p value of the z score for the coefficient (testing whether the estimate is significantly different from zero assuming normality)* ***^c^****95% credible interval generated by taking the 2.5th and 97.5th quantiles of the 100,000 chain iterations produced by the MCMC model* ***^d^****Effective Sample Size (ESS) the number of iterations required to obtain the estimate (independent, identically distributed sample)* **^e^**The mean of the 100,000 chain iterations after converting from the log odds scale to the odds scale. ***^f^****95% credible interval is generated by taking the 2.5th and 97.5th quantiles of the 100,000 chain iterations after converting from the log odds scale to the odds scale.* ***^g^****Bayesian p value: The proportion of the 100,000 chain iterations that were above zero (i.e. odds ratios above one). This provides an estimate of the posterior probability that the odds ratio is above one; although care needs to be taken when all iterations are on the same side, leading to a Bayesian p value of 0 or 1.* | | | | | | | | | |

**Table C.6:** Markov Chain Monte Carlo (MCMC) multilevel model for mixed centres only determining the effect of blinding and whether reader 1 recalls or not on reader 2 recall rate. Reader 1 recall by whether reader 2 is blinded is included as an interaction term. The one-sided Bayesian p values give the proportion of chain iterations which are above zero (i.e. odds ratios above one).

## Appendix D: Tumour Characteristics

Invasive disease was present in 78.4% (2,570/3,277) for blinded and 76.6% (4,503/5,881) for not blinded (χ^2^(1)=4.0, p=0.0449); with no significant evidence of any difference for: disease grade (χ^2^(2)=0.67, p=0.7), the number of positive axillary nodes (χ^2^(2)=3.0, p=0.2), and the mean diameter of the tumour for blinded (16.5mm, standard deviation (SD) 12.6mm) and not blinded (16.2mm, SD 11.8mm; t=1.0, p=0.3). When invasive disease was not present, there was no evidence of an effect of blinding on grade of DCIS (χ^2^(2)=1.99, p=0.37).

|  | | **Reader 2 Blinded** | | **Reader 2 Not Blinded** | | **Hypothesis Tests** |
| --- | --- | --- | --- | --- | --- | --- |
|  | | Count | Percentage | Count | Percentage |  |
| Women |  | 382,490 |  | 736,701 |  |  |
| Cancer detected | Yes | 3,355 | (0.88%) | 6,301 | (0.86%) |  |
|  | No | 379,135 |  | 730,400 |  |  |
| Invasive disease present | Yes | 2,570 | (78.4%) | 4,503 | (76.6%) | χ^2^(1)=4.02 |
|  | No | 707 | (21.6%) | 1,378 | (23.4%) | p=0.04 |
|  | No data | 78 |  | 420 |  |  |
|  | Total | 3,355 |  | 6,301 |  |  |
| Disease grade | 3 | 535 | (20.9%) | 896 | (20.1%) | χ^2^(2)=0.67 |
|  | 2 | 1,371 | (53.6%) | 2,399 | (53.9%) | p=0.71 |
|  | 1 | 651 | (25.5%) | 1,155 | (26.0%) |  |
|  | No data | 13 |  | 53 |  |  |
|  | Total | 2,570 |  | 4,503 |  |  |
| Number of positive axillary nodes | 0 | 1,944 | (76.9%) | 3,466 | (78.5%) | χ^2^(2)=3.00 |
|  | 1,2 | 438 | (17.3%) | 695 | (15.7%) | p=0.22 |
|  | 3+ | 146 | (5.8%) | 255 | (5.8%) |  |
|  | No data | 42 |  | 87 |  |  |
|  | Total | 2,570 |  | 4,503 |  |  |
| Maximum diameter of invasive disease (mm) | n | 2565 |  | 4494 |  | t=1.00 |
|  | Mean (SD) | 16.5 | (12.6) | 16.2 | (11.8) | p=0.32 |
|  | Median | 14 |  | 14 |  |  |
|  | Quartiles | 9 | 20 | 9 | 20 |  |
|  | Min / Max | 0 | 199.9 | 0.4 | 180 |  |
| DCIS grade | High | 387 | (62.9%) | 734 | (60.3%) | χ^2^(2)=1.99 |
|  | Medium | 166 | (27.0%) | 367 | (30.2%) | p=0.37 |
|  | Low | 62 | (10.1%) | 112 | (9.2%) |  |
|  | None | 0 | (0.0%) | 4 | (0.3%) |  |
|  | No data | 92 |  | 161 |  |  |
|  | Total | 707 |  | 1,378 |  |  |

**Table** **D.1**: Tumour characteristics by Reader 2 blinded or not blinded. The hypothesis tests are the χ^2^ test for independence, except for invasive disease present (test for equality of two proportions) and the maximum diameter of invasive disease (t test). For DCIS grade the categories of “Low” and “None” were combined in the χ^2^ test due to small cell numbers.

## Appendix E: Further Test Accuracy Estimates Based on Interval Cancer Data

Interval cancer follow up data was used to provide an approximate assessment of test accuracy statistics for the cohort. Using interval cancers within three years of screening we separated the women not recalled into “false negatives” (women not recalled who had an interval cancer within three years of screening) and “true negatives” (women not recalled and either did not have an interval cancer recorded in their follow up data or did not have follow up data). For consistency within this analysis anyone recalled, had no cancer detected, and had an interval cancer within three years of screen is now classified as a “true positive”, rather than a “false positive”. This is not perfect, since interval cancers are not necessarily cancers missed at screening; some will have developed since the screen but this gives an indication of the sensitivity for a blinded versus not blinded strategy. We performed an equality of proportions test to determine whether these test accuracy metrics were statistically significant.
